# Serotype Chimeric Human Adenoviruses for Cancer Gene Therapy

**DOI:** 10.3390/v2102196

**Published:** 2010-09-30

**Authors:** Tuuli Ranki, Akseli Hemminki

**Affiliations:** 1 Cancer Gene Therapy Group, Molecular Cancer Biology Program, University of Helsinki, P.O. Box 63, 00014 University of Helsinki, Finland; E-Mail: tuuli.ranki@helsinki.fi; 2 HUSLAB, Helsinki University Central Hospital, P.O. Box 100, 00029 HUS, Helsinki, Finland; 3 Haartman Institute & Transplantation Laboratory, University of Helsinki, P.O. Box 21, 00014 University of Helsinki, Finland; 4 Finnish Institute for Molecular Medicine, University of Helsinki, P.O.Box 20, 00014 University of Helsinki, Finland

**Keywords:** adenoviruses, serotype chimerism, neutralizing antibodies, tumor targeting, liver detargeting

## Abstract

Cancer gene therapy consists of numerous approaches where the common denominator is utilization of vectors for achieving therapeutic effect. A particularly potent embodiment of the approach is virotherapy, in which the replication potential of an oncolytic virus is directed towards tumor cells to cause lysis, while normal cells are spared. Importantly, the therapeutic effect of the initial viral load is amplified through viral replication cycles and production of progeny virions. All cancer gene therapy approaches rely on a sufficient level of delivery of the anticancer agent into target cells. Thus, enhancement of delivery to target cells, and reduction of delivery to non-target cells, in an approach called transductional targeting, is attractive. Both genetic and non-genetic retargeting strategies have been utilized. However, in the context of oncolytic viruses, it is beneficial to have the specific modification included in progeny virions and hence genetic modification may be preferable. Serotype chimerism utilizes serotype specific differences in receptor usage, liver tropism and seroprevalence in order to gain enhanced infection of target tissue. This review will focus on serotype chimeric adenoviruses for cancer gene therapy applications.

## 1. Introduction

Significant reductions in cancer mortality have been achieved by improving cancer prevention, early diagnosis and treatments. Nevertheless, incidence rates continue to rise and cancer is already the most common cause of death in many countries, causing approximately 6.7 million deaths annually worldwide [1]. Moreover, traditional cancer therapeutics are associated with undesirable and even life-threatening side effects. Also, significant cross-resistance exists between different chemotherapeutics, underlining the need for novel therapy approaches.

While perhaps only 5% of cancers are hereditary, on the molecular level cancer is always a genetic disease resulting from the accumulation of mutations in various key regulatory genes. Therefore, gene therapy approaches have become a rational focus of interest in developing cancer treatments. Traditionally, cancer gene therapy has aimed at transferring a gene for correction of the disease phenotype or to express therapeutic molecules inside or near the target cell. Virotherapy is a slightly different approach, where the replicating virus itself is the therapeutic agent, incorporating amplification of the therapeutic effect from the initial viral dose. However, oncolytic viruses are often armed with therapeutic molecules to combine the benefits of gene delivery with the more potent oncolytic platform.

Replicating adenoviruses have shown excellent patient safety in clinical trials [2–5], but unfortunately efficacy has been variable, especially in the context of systemic delivery. The success of cancer gene therapy is dependent on the ability of the vector to deliver the therapeutic gene (or viral genome in the case of oncolytic viruses) into the target tissue. Unfortunately, there are various factors that prevent systemically delivered viruses from reaching their target, and therefore extensive research efforts have focused on improving the delivery of adenoviral vectors. In the context of oncolytic virotherapy, genetic engineering is the only feasible means to modify viruses for tropism alteration or expansion, as the whole approach depends on the production of progeny virions which should display the qualities the injected virus displays. The goal of engineering vectors is to create a single-component agent that can efficiently transduce and kill target cells. Serotype chimerism of vectors is a conceptually elegant approach utilizing serotypic differences in receptor usage and serotype-specific immunity. Serotype chimerism allows targeting to receptors distinct from the coxsackie adenovirus receptor (CAR), which is frequently downregulated in advanced tumors. Important goals also include de-targeting of the liver and avoidance of pre-existing neutralizing antibodies.

## 2. Factors affecting the outcome of systemic delivery of Ad5

There are over 50 different serotypes of adenoviruses that were originally classified depending on their ability to neutralize serum to cross-block them. They can be further divided into six different subgroups A-F based on their ability to agglutinate erythrocytes of different species and on their oncogenicity in rodents (adenoviruses are not oncogenic in humans). Viruses from different subgroups have distinct tropisms, implying distinct receptor usage. Group C adenoviruses, including serotype 5 adenovirus (Ad5), the most widely utilized serotype in cancer gene therapy, utilize the coxsackie-adenovirus receptor (CAR) as a primary attachment molecule [6,7]. Later it was demonstrated that also group A, D, E and F adenoviruses use CAR [7], though not all of them as a primary receptor [8,9]. The expression levels of CAR correlate with the susceptibility of a particular cell type to Ad infection [10]. Loss of CAR expression correlates with tumor progression [11], resulting in low expression in advanced disease and subsequently variable gene transfer efficacy. Moreover, Ad5 vector biodistribution *in vivo* does not seem to depend on CAR distribution [12]. Various blood coagulation factors, such as vitamin K-dependent factors VII, IX and X (FVII, FIX and FX, respectively) as well as complement protein C4BP have been shown to play a role in tropism by bridging adenovirus to cell surface receptors, especially on hepatocytes and liver Kupffer cells (KCs) [13–16]. When treating metastatic disease, systemic delivery would be appealing and therefore liver tropism might be a major barrier for the systemic use of Ad5 based vectors [17–19]. However, these aspects are only known in mice and it is unclear if they accurately represent the human situation. Neutralizing antibody (NAb) response to Ad5 is also a major determinant of the fate of systemically delivered virus [20,21], which may be relevant as the majority of humans have been exposed to Ad5 and many have circulating Ad5-specific NAbs.

### 2.1. Tumor targeting by fiber serotype chimerism

**Figure 1:**
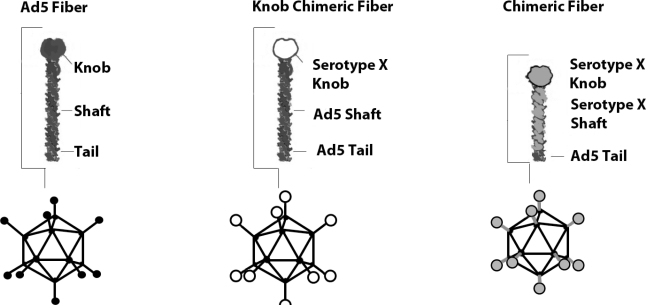
The principal of serotype chimeric fiber modifications. Depicted from left to right are the wild type Ad5 capsid, Ad5 capsid featuring the knob from a distinct serotype and Ad5 capsid featuring the shaft and knob from a different serotype.

Various Ad5 capsid locales have been genetically modified to enhance tumor transduction. The fiber knob domain is principally responsible for binding CAR, and thus the Ad5 fiber protein has been the primary focus of genetic modification [22]. Different serotypes have significant similarities in the fiber architecture [23,24] which allows the creation of chimeric vectors, where the whole fiber or only the knob region is switched to that of another serotype (Figure 1). Fiber chimeras using non-CAR binding knobs display CAR independent transduction and recognize alternate receptors.

#### 2.1.1. Fiber chimerism

Several groups have studied the feasibility of fiber chimerism in terms of viral tropism alteration (Table 1). Even before CAR was identified as the primary receptor for Ad5 in 1997 [6], the replacement of its fiber with that of Ad7 was shown to be feasible due to homology in the fiber tail regions [25]. Ad7 fiber expanded the tropism of the chimeric Ad5/7 vector towards a distinct receptor and allowed infection of CAR deficient dendritic cells (DCs). Subsequently, it was shown that Ad7 and other group B adenoviruses utilize either CD46 or receptor X as the primary attachment molecule instead of CAR [26,27]. The subdivision of subgroup B viruses into species B1 (serotypes 3, 7, 16, 21 and 50) and B2 (serotypes 11, 14, 34 and 35) was based on genetic differences [28]. CD80 and CD86 have also been proposed as receptors for species B adenoviruses [29,30]. Tuve *et al.* suggested a revised classification of subgroup B viruses based on their receptor usage [27]. According to this division, group I serotype B adenoviruses utilize CD46 (serotypes 16, 21, 35 and 50), group II adenoviruses utilize receptor X (serotypes 3, 7p and 14), and group III Ad11p utilizes both.

Subgroup B viruses do not use CAR, and therefore the efforts to construct serotype chimeric viruses have largely focused on exploiting them, though viruses from all subgroups have been utilized (Table 1). To this end, replacing the Ad5 fiber with that of subgroup B Ad35 resulted in enhanced infectivity of hematopoietic stem cells [31]. More importantly, wild type Ad35 displayed efficient *in vitro* cytotoxicity in prostate, breast, liver and ovarian cancer cells [32]. When mice bearing subcutaneous prostate cancer tumors were treated intratumorally or intravenously with wild type Ad35, however, no significant anti-tumor effect was witnessed, nor was there any impact on survival. The authors concluded that expression levels and accessibility of the primary receptor CD46 is not the sole determinant of Ad35 infectivity and anticancer activity *in vivo*.

The fiber from another subgroup B virus, Ad16p, was found to confer enhanced gene transfer by Ad5 based vectors to cardiovascular and synovial tissues [33]. Importantly, a recent study demonstrated efficient transduction of brain tumor cell lines and CD133+ “tumor initiating cells” by Ad16p [34]. Another subgroup B virus, Ad11p, was shown to efficiently infect and kill prostate, breast, liver and ovarian cancer cells *in vitro* [32]. Furthermore, chimeric Ad5/11p was shown to kill oral and esophagus cancer cells *in vitro* [35]. Importantly, systemically delivered wild type Ad11p resulted in a significant survival benefit in the same murine model of prostate cancer, where Ad35 failed [32].

#### 2.1.2. Knob chimerism

Substitution of the knob region instead of the entire fiber is a variation of the serotype chimerism approach. One such virus is the serotype 3 chimeric Ad5/3, which has the Ad5 fiber knob replaced by Ad3 knob [36,37]. Retaining the Ad5 fiber shaft may be beneficial, as it has been shown that the length of the shaft plays a crucial role in adenoviral infectivity [38]. The long Ad5 shaft may be superior to a short shaft, in the context of the Ad5 backbone, as the shorter shaft might result in charge-dependent repulsion between exposed negatively charged Ad5 hexon loops and the acidic cell-surface. Therefore, changing only the knob region but retaining the long Ad5 shaft is an appealing approach to retarget the virus from CAR but to take advantage of the favorable effect the longer shaft length may have on infectivity. Indeed, Ad5/3 chimeric virus has been shown to display enhanced gene transfer in various cancer models, including prostate cancer, ovarian cancer, breast cancer, gastric cancer and renal cancer [37,39–43]. Enhanced gene transfer efficacy was translated into improved cell killing efficacy with an oncolytic Ad5/3-Δ24 in an orthotopic murine model of ovarian cancer [44]. Furthermore, Ad5/3-Δ24 and its various tissue specific promoter controlled variants displayed effective antitumor activity against tumors derived from CD44^+^/CD24^−/low^ breast cancer initiating cells [45,46]. Ad5/3-Δ24 has also been tested in several other tumor types with impressive efficacy results [39,41–44,47]. In addition to a chimeric fiber, Ad5/3-Δ24 features a 24 base pair deletion in *E1A* conserved region 2 (CR2). Dysfunctional E1A is incapable of binding and releasing transcription factor E2F1 from retinoblastoma (Rb), leading to attenuated replication in quiescent normal cells. The Rb/p16 pathway is universally disrupted in cancer cells [48], and, thus, free E2F1 is abundant in cancer cells, allowing replication of Δ24-viruses in most solid tumors.

### 2.2. Liver detargeting by serotype chimerism

The liver is the most relevant organ with regard to adenovirus associated toxicity as corroborated by a fatality in an adenoviral gene therapy trial [54]. Adenoviruses can cause severe toxicity in immunocompromised individuals [55]. In mice, Ad5 displays liver tropism that adversely affects its availability after systemic delivery [17,19]. In humans, the biodistribution of adenovirus is not known.

Hepatocyte transduction ablation has been attempted with various capsid modifications. Smith and colleagues created a mutation in the Ad5 HSPG binding fiber shaft KKTK motif that resulted in reduced liver tropism in mice, rats and non-human primates [56,57]. Various attempts to modify *in vivo* tropism by abolishing CAR interactions have failed in avoiding the liver tropism of Ad5 [58,59]. Furthermore, Ad5 knob replacement with serotype 3 knob does not significantly alter Ad5 liver tropism, though CAR binding is abrogated [39,41–43], implying that the presence of CAR is not a critical factor in determining the susceptibility of tissues to adenovirus *in vivo*. Blood factors serving as a bridge between the virus and liver cells may allow efficient liver transduction despite CAR binding ablation [13,14]. Hexon has been suggested to have a major role in Ad5 liver tropism and recent studies have clarified the high-affinity interaction of FX with hexon in FX-mediated liver gene transfer [16,60]. This suggests that using viruses based on other serotypes with weaker binding between hexon and coagulation factors would reduce liver tropism. Indeed, other serotypes than Ad5 conferred decreased FX binding to hexon, with subgroup B Ad35 and Ad3 binding FX weakly and subgroup D Ad48 and Ad26 not binding FX at all [16,61]. Hexon chimeric Ad5/Ad6-gag and Ad5/Ad12-gag vectors were not neutralized with Ad5 NAbs, suggesting that hexon contains the major antigenic determinants for NAbs [62]. However, the hexon chimerism approach seemed feasible only when the hexon switch was performed within the same subgroup, suggesting lack of adequate structural similarity between different subgroups. Recently, it has been shown that mutation of critical amino acids from two hypervariable regions in hexon eliminates FX binding and subsequent FX-mediated liver gene transfer in mice [63], which further highlights the role of hexon in liver tropism of Ad5 based vectors.

However, contradicting results were obtained when *in vivo* phage display and capsid engineering were combined to develop a renal-targeted Ad5/19p-HIT vector, which had been proposed to be promising for systemic delivery to renal tumors [52,64]. This vector displayed lower FIX and FX binding and reduced hepatic tropism, and, importantly, favorable tumor-to-liver transduction ratios in murine models of renal cancer when compared to Ad5 even though it featured intact Ad5 hexon. These results suggest that the role of Ad5 fiber in liver tropism should not be overlooked. It has been postulated that interactions between FIX and fiber [64] or the length of the shaft may also play major roles in hepatocyte transduction. Shortness of the shaft has previously been reported to affect liver tropism of fiber modified vectors [49] and the Ad19p shaft is the same length as Ad35 shaft [65]. The effect of shaft length is corroborated by a finding that subgroup C Ad6 does not induce liver toxicity in mice [32] although its hexon binds to FX with the same efficiency as Ad5 [16]. Ad6 fiber shaft is three β-turn repeats shorter than that of Ad5 [66].

### 2.3. Avoidance of pre-existing immunity by serotype chimerism

Human Ads have been classified into over 50 serotypes based on lack of cross-neutralization. The majority of humans have been exposed to Ads, resulting in immunological memory. Also, 40–97% of the human population present neutralizing antibodies (NAbs) against Ad5 [67]. Adenovirus NAb titers vary by detection method and geographic location and are higher in non-US and non-European countries [68]. NAbs bind to capsid proteins and block Ad internalization by target cells, and elicit induced uptake to Fc-receptor bearing immune cells such as kupffer cells (KCs) and dendritic cells (DCs), resulting in rapid vector clearance and inflammatory responses [69,70]. Innate immune responses and pre-existing immunity against Ads are major determinants of the fate of systemically delivered Ads [20,21], and although pre-existing immunity does not seem to adversely affect the antitumor efficacy of locally delivered Ad [71], high NAb titer may compromise systemic delivery [72]. Furthermore, in naïve individuals, NAbs develop against viral capsid proteins within weeks after virus encounter thus complicating systemic re-administration.

The first serotype chimeric vector reported, Ad5/7, displayed unaltered neutralizing immune responses, suggesting that NAbs generated against fiber do not constitute a significant fraction of the total NAb population. Indeed, the seroprevalence of rare human Ads is significantly lower than of Ad5, with less than 7 % of humans presenting NAbs against Ad35, for example [73].

Since the bulk of neutralizing antibody response is directed against hexon [74], the most abundant capsid protein, fiber chimeric Ad5 vectors may still face the problem of pre-existing immunity. There are contradicting studies, however, that suggest a significant role also for anti-fiber NAbs in determining the success of gene transfer *in vivo,* especially in the context of low NAb titers [75]. For example, an Ad5/3 [36] vector featuring serotype 3 knob was shown to achieve effective gene transfer in Ad5 pre-immunized mice, perhaps highlighting the crucial role of knob in mediating infection [75]. A study corroborating the importance of anti-fiber antibodies showed that an Arg-Gly-Asp (RGD) modification in the HI-loop of Ad5 fiber allowed partial escape from NAbs present in the ascites fluid [76]. Moreover, anti-fiber antibodies recognize conformational epitopes of the trimeric form of the fiber, and thus even modest changes to the knob may cause critical alterations in the three-dimensional structure, thus avoiding antibody recognition [77]. The putative requirement of anti-fiber antibodies for productive synergism with other NAbs may be important.

## 3. Clinical Use of Serotype Chimeric Viruses

Only a few chimeric viruses have entered clinical use. All published data are derived from the use of two replicating viruses, Ad5/3-COX2L-Δ24 and Ad5/3-Δ24-GMCSF, both of which feature the serotype 3 knob in an Ad5 backbone [53,78–80] (Table 2). Both viruses harbor a 24 bp deletion in *E1A CR2*, targeting viral replication to cells with a defective Rb/p16 pathway. Furthermore, Ad5/3-COX2L-Δ24 also features a cyclo-oxygenase 2 promoter (COX2L) controlling the expression of E1A, and Ad5/3-Δ24-GMCSF has the granulocyte macrophage colony stimulating factor (GMCSF) gene inserted into the E3 region to evoke antitumor immunity. Previously, a GMCSF-producing oncolytic virus (Ad5-D24-GMCSF) with a wt capsid was shown to induce tumor-specific immunity in an immunocompetent syngeneic hamster model semipermissive for human adenoviruses [81]. Prior to clinical studies, also the chimeric Ad5/3-Δ24-GMCSF was studied for selectivity and efficacy in immunocompetent hamsters [53]. The virus was effective in hindering the growth of aggressive syngeneic pancreatic tumors, with and without low-dose cyclophoshamide used to reduce regulatory T-cells. Tumor-selective replication and transgene expression was demonstrated by measuring an increase in viral copy number and GMCSF production in intratumorally injected tumors, while there was no increase in either in the liver.

Patients with various cancer types and advanced solid tumors were treated with Ad5/3-COX2L-Δ24 and Ad5/3-Δ24-GMCSF with excellent safety [53,79,80]. Mostly mild, grade 1–2 symptoms were encountered with total viral doses of up to 3 × 10^11^ VP. Objective evidence of antitumor activity ranging from 61% to 67% was seen after a single round of treatment [53,80]. Specifically, Ad5/3-COX2L-Δ24 treatments resulted in one partial response and one minor response in five of the radiologically evaluable patients [79,80]. Several other patients had decreases in tumor markers and overall, 11/18 patients had evidence of anti-tumor activity. An increase in viral copy number was seen in the serum of 11/17 evaluable patients two days or later post treatment compared to day 1, suggesting viral replication. Rapid induction of Ad5/3 NAbs was observed in patients within three weeks of treatment and titers remained high for several weeks. High NAb titers, however, did not prevent Ad5/3-COX2L-Δ24 from replicating in tumors as viral genomes and increasing NAb titers could be measured in blood concurrently in many patients.

Treatment with Ad5/3-Δ24-GMCSF resulted in objective clinical benefit in 8/12 patients as evaluated by radiology with Response Evaluation Criteria In Solid Tumors (RECIST) criteria [53]. Increases in the blood titer of Ad5/3-Δ24-GMCSF when compared to day 1 were seen in 8/15 patients, suggesting viral replication. NAbs increased in all patients, but no correlation was seen between NAb titers and viral dose, virus shedding in blood, antitumor activity, or toxicity. Increases in CD8^+^ lymphocytes against Ad5 were seen, suggesting that adaptive cellular responses can be produced even in patients with advanced disease. Furthermore, CD8^+^ cells against a tumor epitope survivin increased in the majority of the patients, suggesting that an antitumor immune response was also evoked.

## 4. Conclusions

Ad5 is the most commonly used platform for the development of oncolytic adenoviruses for the treatment of cancer. Serotype chimerism may be a useful means to increase efficacy of the approach. It can be utilized to circumvent the dependence on CAR and influence the liver tropism of viruses, although not all reports agree on the latter. Also, the type of chimera may determine the presence or absence of an effect on mouse liver tropism. Chimeras may also be useful for avoiding pre-existing serotype specific antibodies and some chimeras may be less immunogenic than others. The importance of hexon/FX interactions in liver tropism have been demonstrated [16,63], indicating that swapping merely the Ad5 fiber to that of another serotype would not result in abolished liver tropism. Furthermore, FX binding is postulated to be specifically associated with the hexon hypervariable regions [63], suggesting that small modifications in hexon amino acids might eliminate liver transduction. Furthermore, the role of Ad5 fiber in liver tropism via binding to FIX [64] and through the KKTK interaction with heparin sulfate proteoglycans (HSPG) [56] should not be underestimated. NAb responses against various capsid proteins seem relevant in determining the fate of systemically delivered adenoviruses, thereby encouraging further development of serotype chimeric vectors. Such viruses could also feature modifications in the hexon hypervariable region if Ad5 is used as the platform for vector development. For assessing the relevance of preclinical studies, it would be key to understand biodistribution and the presence or absence of adenevirus liver tropism in humans.

## Figures and Tables

**Table 1:** Selected serotype chimeric adenoviruses.

**Virus**	**Subgroup**	**Capsid modification**	**Receptor / Specific homing**	**Results**	**Ref.**
Ad5/7	C/B1	Ad7 fiber	CD46	infection of CAR deficient DC	[25]
Ad5/35	C/B1	Ad35 fiber	CD46	Infection of CAR-deficient CD34+ hematopoietic stem cells.	[31]
Ad5/35S	C/B1	Ad35 fiber	CD46	Hepatocyte transduction was independent of the interaction with CAR and reduced 10 fold with short shaft.	[49]
Ad5/35L	C/B1	Ad35 knob	CD46		
Ad5/9S	C/C	Ad9 fiber	CAR		
Ad5/9L	C/C	Ad9 knob	CAR		
Ad5.Fib12	C/A	Ad12 fiber	CD46	Increased transduction of patient-derived glioma cells with serotype 35, 16, 50 and 11 chimeras.	[50]
Ad5.Fib16	C/B1 (I)	Ad16 fiber	CD46		
Ad5.Fib35	C/B1 (I)	Ad35 fiber	CD46		
Ad5.Fib50	C/B1 (I)	Ad50 fiber	CD46		
Ad5.Fib7	C/B1 (II)	Ad7 fiber	receptor X		
Ad5.Fib11	C/B2 (III)	Ad11p fiber	receptor X and		
Ad5.Fib10	C/D	Ad10 fiber	CD46		
Ad5.Fib17	C/D	Ad17 fiber	sialic acid		
Ad5.Fib24	C/D	Ad24 fiber	sialic acid		
Ad5.Fib30	C/D	Ad30 fiber	sialic acid		
Ad5.Fib33	C/D	Ad33 fiber	sialic acid		
Ad5.Fib37	C/D	Ad37 fiber	sialic acid		
Ad5.Fib38	C/D	Ad38 fiber	sialic acid		
Ad5.Fib47	C/D	Ad47 fiber	sialic acid		
Ad5.Fib40S	C/F	Ad40 short	sialic acid		
		fiber	unknown		
ColoAd1 (oncolytic)	B1/B2 (II)/(III)	major capsid proteins from Ad11p	receptor X and CD46	Directed evolution resulted in an Ad11p virus with a nearly complete E3 region deletion, smaller deletion in E4 and a chimeric Ad3/Ad11p E2B region. Over 2 log increase in potency and selectivity when compared to ONYX-015 on colon cancer cell lines and *in vivo.*	[51]
Ad5/3luc1	C/B1	Ad5 fiber, Ad3 knob	receptor X	Enhanced gene transfer to various cancer cell lines and primary tumor tissues.	[37,39,41–43]
Ad5/3-Δ24 (oncolytic)	C/B1	Ad5 fiber, Ad3 knob	receptor X	Enhanced cell killing of cancer cell lines and xenograft tumors.	[39,41–44]
Ad5/19p-HIT	C/D	Ad19p fiber, HIT peptide	sialic acid, phage display-selected for homing to kidneys	Enhanced gene transfer to renal cancer cell lines and xenograft renal cancer tumors *in vivo*, decreased gene transfer to liver	[9,52]
Ad5/3-Δ24-GMCSF	C/B1	Ad5 fiber, Ad3 knob	receptor X	Enhanced cell killing of cancer cell lines and syngeneic hamster tumors. Objective clinical benefit in 8/12 patients with progressing chemotherapy refractory solid tumors as evaluated by radiology with RECIST criteria	[53]

**Table 2:** Clinical use of serotype chimeric viruses.

**Virus**	**Capsid modification/Other modifications**	**Cancer/Patients (N)**	**Highest dose**	**Delivery route**	**Results**	**Ref.**
Ad5/3-COX2L-Δ24	Ad5 fiber, Ad3 knob / the *Cox2*-promoter contolling *E1A* with a 24bp deletion	Progressing chemotherapy refractory solid tumors/18	3 × 10^11^ VP	i.v., i.t.’ i.c.	Antitumor activity in 61% of patients; measured according to RECIST criteria or as tumor markers	[80]
Ad5/3-COX2L-Δ24	Ad5 fiber, Ad3 knob / the *Cox2*-promoter contolling *E1A* with a 24bp deletion	Chemotherapy refractory high risk non-4-S, stage 4 neuroblastoma with lymph node metastasis/1	1 × 10^11^ VP	i.t., into lymph node, i.v.	Primary tumor regression by 71%, complete response in bone marrow.	[79]
Ad5/3-Δ24-GMCSF	Ad5 fiber, Ad3 knob/*E1A* with a 24bp deletion, GMCSF in E3	Progressing chemotherapy refractory solid tumors/21	4 × 10^11^ VP	i.t, 1/5 of the dose i.v.	Antitumor activity in 67% of evaluable (12) patients by radiological assessment, reduction of tumor marker values in 2 patients. Resolution of ascites and pleural effusion formation in 2 patients.	[53]

i.v. = intravenous; i.t. = intratumoral; i.c. = intracavitary
